# Cultural differences in stress and affection following social support receipt

**DOI:** 10.1371/journal.pone.0256859

**Published:** 2021-09-09

**Authors:** Vida Pourmand, Kendall A. Lawley, Barbara J. Lehman

**Affiliations:** Department of Psychology, Western Washington University, Bellingham, Washington, United States of America; Iwate Medical University, JAPAN

## Abstract

Culturally appropriate social support predicts better psychological outcomes. Motivation for providing social support may vary cross-culturally, with more independent cultures valuing self-esteem and more interdependent cultures valuing closeness. Participants in the U.S. (N = 85) and Singapore (N = 78) reported on emotions and social support receipt using the Day Reconstruction Method. We examined cultural differences in stress and affection, and tested country as a moderator of the associations between both social support receipt and social support motivation, and next-episode emotions. Multilevel modeling analyses showed that not only did the emotional correlates of social support receipt vary by country, but that recipient perceptions of esteem-building and closeness-fostering SS also differentially correlated with subsequent emotion. For example, esteem-building SS predicted greater next-episode stress for Singaporean participants, but less stress in the U.S. Esteem-building SS predicted more next-episode affection only in the U.S. Culturally appropriate social support predicts positive psychological outcomes. This research highlights the importance of considering culture when examining the dynamic emotional correlates of social support receipt.

## Introduction

Socially supportive relationships promote positive emotions and help to decrease the negative consequences of stress. Social support (SS) receipt can be understood as contact from a friend or loved one that allows the recipient to know they are cared for and are part of a mutually beneficial social network that provides comfort and help in times of stress [1]. Receiving SS is an effective buffer against a range of negative physical and psychological responses to stress and adversity [1]. SS predicts better health and is a buffer against the negative effects of stress [1]. In turn, SS receipt can promote feelings of affection and social connectedness and reduce feelings of social isolation. SS is a consistent predictor of well-being and culturally appropriate SS can promote positive psychological outcomes [2, 3]. Because cultural context likely shapes the experience of receiving SS, it is important to consider the role of culture in SS processes.

### Socially supportive interactions

Socially supportive interactions can take many forms. SS may promote problem solving, thereby helping the recipient to change the situation that is causing the stress [1]. SS may also provide emotional reassurance making the recipient feel that they are loved and cared for, perhaps by providing physical comfort such as hugging or hand holding [1]. It is important to distinguish among the concepts of enacted social support, perceived SS, and received support. Enacted support examines the actual exchange of supportive communications between provider and recipient, while perceived support is focused on the recipients’ perceptions of the characteristics of available support [4]. Perceived network support includes believing support is available during difficult times. Finally, this study focuses on received SS, which considers the recipient’s perception of the extent to which they received SS from another person [4]. Further, each of these support concepts encompasses many different forms of support (e.g. emotional support versus instrumental support [3]). This study does not distinguish among different forms of support, but rather focuses on cultural differences in the emotional consequences of received SS that functions either to foster closeness or to build self-esteem.

Although the concepts of closeness-fostering and esteem-building SS have typically been studied in the psychological literature from the perspective of SS provider motivations [5] it is equally, if not more, important to consider how SS recipients perceive SS interactions. Previous research in the supportive communication literature shows that the recipient’s perceptions of help, encouragement, and support they received greatly influences the outcomes of a SS interaction [6]. This is important because the provider’s SS experience does not necessarily mirror the recipient’s [7]. SS interactions involve a complex combination of factors including the relationship between the provider and the recipient, the type of supportive message being conveyed, and the context in which support is given [8]. Multiple factors therefore influence the effectiveness of a SS interaction and the subsequent outcomes of that interaction [8]. Specifically, a provider may intend to promote closeness or provide comfort to a loved one, but the SS recipient may not perceive those intentions. Even if a SS provider believes they are providing effective SS, if the SS recipient does not perceive their SS partner as being appropriately supportive the SS will not be as beneficial [9]. Ultimately, the recipient’s perception of the interaction may be the best indicator of whether a SS interaction is successful.

Prior cross-cultural research has highlighted two potential motivations underlying SS provision. Specifically, Chen and colleagues [5] compared the motivations of European American and Japanese SS providers. Motivation for fostering closeness with the SS recipient predicted SS provision in both Japan and the United States (U.S.), but motivation for bolstering the self-esteem of the SS recipient only predicted SS provision in the U.S. Additionally, H. S. Kim and colleagues found that European Americans purposefully sought out SS to cope with daily stressors more than Koreans, and that Koreans reported SS involving feeling connected to a social network more than European Americans [10]. Taken together, these findings suggest that culture may moderate SS processes. This study examines the role of socially supportive interactions in promoting everyday feelings of affection and stress. In contrast to the work of Chen and colleagues, we examine recipient perceptions of the success of received SS in building esteem and closeness, and the consequences of those transactions for subsequent reports of affection and stress.

### Culture and social support receipt

Because SS is an important contributor to well-being that involves interacting with others, it is necessary to view SS interactions through a cultural lens [2]. Some cultural differences can be understood as drawing on more independent or interdependent values. In more independent cultural contexts, such as the U.S., people are more likely to view themselves as unique and separate, and individuality and self-expression are especially important to the self-concept [11]. In this cultural context, people typically value high self-esteem, are motivated to retain a sense of personal influence, and desire to be in charge of their personal life circumstances. Because of this, one important goal of SS provision in independent cultural contexts may be to build the self-esteem of the SS recipient [5]. In more interdependent cultural contexts, such as in Singapore, people tend to perceive themselves in relation to others and to view connection to the broader social group as a primary goal of healthy social functioning [11]. Because group cohesion is important in this context, a goal of SS in interdependent contexts is often to foster relationship closeness and group connectedness [5]. The present study therefore explores the psychological consequences of SS receipt motivated by self-esteem and by closeness in the more independent cultural context of the U.S. and the more interdependent cultural context of Singapore.

Evidence suggests there are important cultural differences in the role of self-esteem in personal self-construal and these differences have implications for SS receipt. Campbell found that self-esteem was the largest predictor of life satisfaction for a national sample of adults in the U.S. [12]. However, that study was conducted in the U.S., which is a largely independent culture. Diener and Diener found that the correlation between self-esteem and life satisfaction was attenuated in more interdependent nations in comparison to more independent nations [13]. This difference may be due to the fact that people in more independent cultural contexts consider individuality and self-expression to be essential to self-concept [11]. Those in more independent contexts therefore tend to emphasize high self-esteem and a strong sense of self. For these reasons, when it comes to SS provision in more independent cultural contexts, an important goal can be to cultivate the self-esteem of the SS recipient [5]. Not only is esteem-building the goal of the provider, but SS recipients of European American background are motivated to build their self-esteem when they seek out explicit SS in response to stress [14]. Self-esteem can be promoted through SS that makes the SS recipient feel valued and accepted for who they are [1, 15]. Such socially supportive interactions are likely to reduce recipient stress and foster feelings of affection in a more independent context. This pattern would be expected if the recipient feels supported and cared for in a culturally appropriate way. In contrast, in interdependent cultural contexts, individuals are encouraged to assimilate to, or become part of, their social networks [16] and expressing high self-esteem would be considered a sign of maladjustment [13]. Therefore, receiving SS intended to boost self-esteem in a more interdependent context may bring about feelings of stress and minimize feelings of affection because it could create an awkward and culturally inappropriate SS interaction. It is therefore important to examine how self-esteem building contributes to recipient psychological outcomes.

Much like self-esteem, evidence suggests that the degree to which SS transactions build interpersonal closeness differs by cultural context. Those from independent and interdependent cultural contexts have different views of how the self is perceived, and these views play a role in how close people feel to those around them [11]. In more independent cultural contexts, where individualism and personal expression are more likely to be emphasized, interpersonal closeness tends to be less important [11]. For example, a study of cohabiting couples in the U.S. found that SS receipt predicted both feelings of closeness and more negative emotions [17]. However, SS receipt affected people differently, such that those who experienced more negative mood following SS receipt also experienced relatively fewer feelings of closeness. Gleason and colleagues suggested this result may reflect the reality that SS receipt can promote feelings of inefficacy. Although cultural differences were not tested in the preceding studies, social assimilation is more important in interdependent cultural contexts [11]. Desire for social connectedness may motivate socially supportive feelings of closeness that allow the individual to feel connected to their support provider [11]. This study examines how SS perceived as fostering interpersonal closeness predicts the psychological outcomes of stress and affection for SS recipients in the U.S. and Singapore.

### Culture and the implications of social support for stress and affection

The emotional correlates of effective SS processes likely also vary by culture. Feelings of stress not only typically precipitate the need for SS, but are also likely to be reduced following the receipt of culturally appropriate SS. In contrast to stress, which is individually experienced, feelings of affection are grounded in interpersonal dynamics. It is important to consider the contrasting emotional correlates of stress, which can be considered a socially disengaging emotion [18], and affection, which can be considered a socially engaging emotion [18]. Socially engaging emotions stem from pursuing social harmony, which more commonly occurs in more interdependent cultural contexts, whereas socially disengaging emotions are founded in independence and self-promotion [18], a more common occurrence in more independent cultural contexts. This research considers the consequences of socially supportive interactions for subsequent feelings of stress and affection.

Stress is a part of everyday life regardless of culture but can have distinct correlates across cultural contexts [19]. For example, Taylor et al. suggest that culture helps to define how people seek SS during stress [20]. Asian Americans have been found to be less likely to reach out to their social networks during times of stress than more independent European Americans. Further, culturally inappropriate forms of SS may actually exacerbate stress [21]. Taylor and colleagues found that European Americans reported greater stress and had higher levels of the stress-linked hormone cortisol during a stressful laboratory task when they imagined a group of people they felt close to, but were not able to ask for SS [21]. However, Asians and Asian Americans reported more stress and had greater cortisol during the stressful task when imagining using SS in the form of advice seeking or talking about their emotions. The current study builds on these findings to investigate whether SS receipt shapes later feelings of stress, and whether these patterns differ by culture or by the motivation underlying the SS.

Feelings of affection should also be considered in a cultural context. Feelings of connectedness can foster affection, and relational partners who are closer and more comfortable with each other tend to report more feelings of affection [22]. To our knowledge, little research has examined cultural differences in the relationship between SS and affection. However, in a study of culture and emotional outcomes following SS provision, Lawley et al. found that SS providers reported more feelings of affection at times when they provided emotional SS [7]. This pattern was observed regardless of cultural context. Additionally, participants in Singapore reported relatively greater feelings of affection overall. This result is important because not only may there be cultural differences in the motivation and experience of SS, but there may also be cultural differences in affection. For this reason, we will be investigating cultural differences in SS and SS processes on later feelings of affection.

### SS processes and cross-situation spillover

When addressing the consequences of SS exchanges for later feelings of stress and affection, it is important to address the concept of spillover. Spillover refers to the process by which social interactions and experiences in prior settings can influence the experience of subsequent events [23, 24]. Lingering negative emotions from prior events can have important consequences for physical health. For example, Leger and colleagues found that participants experienced heightened negative affect that lingered the day after they experienced a minor stressor, and that those with more lingering negative affect experienced more chronic health conditions and greater functional impairment 10 years later [25].

Daily experiences are shaped not only by culture, but also by previous SS interactions and current interpersonal and environmental dynamics. For instance, effective supportive assistance from a coworker in the workplace may help put a support recipient in a better mood when they return home to their family. The feelings associated with that SS interaction do not necessarily dissipate, but rather may help to shape later interactions [24]. Although the subsequent family social dynamics may themselves elicit feelings of affection or stress, the interest in the current study is in the extent to which prior SS experiences may spill over to amplify or buffer later emotional experiences. Spillover from the workplace’s positive SS exchange could affect interpretations of social interactions and experiences later in the day, promoting less stress and more affection.

Previous diary studies examining social interactions have demonstrated spillover in a variety of social exchanges including parent-child social exchanges [26, 27], social exchanges between romantic partners [28], and social exchanges between roommates and friends [29]. This research on spillover suggests that daily social experiences are not isolated incidents, but rather that they can shape subsequent emotions and interactions throughout the day. Receiving SS can result in positive emotional outcomes, such as reduced feelings of stress. However, receiving ineffective or inappropriate SS may actually exacerbate stress and other negative emotional outcomes. Furthermore, given the influence of culture on SS processes [30], it is also important to use a cultural lens when examining the link between previously received SS and subsequent emotions. Within a given cultural context, socially supportive interactions may 1) decrease stress and increase affection, potentially resolving the issue that required SS as well as building interpersonal closeness and affection or 2) increase stress and decrease affection if the SS is inappropriate, unskillful, or insufficient to address situational needs. This study therefore examines the influence of closeness-fostering and esteem-building SS on subsequent stress and affection as a function of culture.

### The present study

The present study explores potential cultural differences in stress and affection for SS recipients following instances of received SS that the recipients believed fostered relational closeness or built personal self-esteem. Specifically, using the Day Reconstruction Method [31], we examine whether SS receipt among college students in Singapore and the U.S. predicted subsequent stress and feelings of affection. The Day Reconstruction Method is optimal for probing the cultural dynamics of SS receipt because it allows for micro-longitudinal tests of whether prior experiences and SS motivations predict subsequent emotions [32]. We hypothesize that:

1. Participants from Singapore will report more stress following SS receipt, in comparison to participants from the U.S.
2. Culture will moderate SS processes, such that those from Singapore would experience more closeness, while those from the U.S. would experience more esteem-building.
3. Culture will moderate psychological outcomes of stress and affection such that SS recipients from the U.S. would report less subsequent stress and more affection if they experienced esteem-building SS in contrast to SS recipients in Singapore.
4. Culture will similarly moderate psychological outcomes of stress and affection such that SS recipients from Singapore would be expected to report less subsequent stress and more affection if they experienced closeness-fostering SS in contrast to SS recipients in the U.S.

## Method

### Participants

163 University students recruited from Psychology subject pools in Singapore (47.9%) and the U.S. (52.1%) participated in this study and received research credit for their participation. The Singaporean sample was 71.6% women and 28.4% men. The U.S. sample had a similar gender ratio with 72.2% women and 27.8% men. The mean age was 21.90 (*SD* = 1.50) for the Singaporean sample and 21.49 (*SD* = 3.41) for the U.S. sample. The Singaporean sample was collected from an urban private University and the U.S. sample was collected from a mid-sized public regional university in a small city in the Pacific Northwest. Despite these differences, no statistically significant differences in income or age were found between the two countries, though the sample from the U.S. exhibited more variability in age.

Although 178 undergraduate students participated in the current study, 176 had viable data for analysis of SS receipt. Data from one participant were removed because errors in data collection made it impossible to match their provided responses on the two days of the study. Additionally, another participant only completed part of the study and was therefore excluded. For analyses that examined SS receipt processes specifically, it was only possible to use reported SS; 13 participants did not report receiving SS.

### Procedure

Participants completed two different hour-long sessions in on-campus computer labs in the U.S. and Singapore. Participants took part in the study on either a Tuesday/Wednesday or a Saturday/Sunday to ensure all participants were reporting on one weekday and one weekend day. Participants used the web-based Qualtrics platform to respond to questions and to provide informed written consent. The survey was administered in English in both locations. This research was approved by the institutional review boards of Singapore Management University (IRB17-007-A001-117) and Western Washington University (secondary review). All participants were asked to think back to their previous day and to conceptualize it as a series of episodes. Using Kahneman and colleagues’ Day Reconstruction Method (DRM) [31] participants were asked to indicate the start and stop times of each episode, provide some descriptive information about each episode, and to indicate whether they received or provided SS during the episode. The concept of SS was defined for participants and some examples were provided. This methodology is useful because it allows researchers to ask participants questions that are only relevant to the research, and it allows for time-lagged outcomes that would otherwise be difficult to capture with traditional self-report data [32]. After completing the diary sheet by describing events from the previous day as episodes, participants indicated to a research assistant that they were finished with the survey and were ready to begin the Qualtrics questions related to SS provision and receipt. Next, participants responded to questions describing characteristics of each episode from the prior day. In addition to the measures described below, participants indicated their main activities and social interactions for each episode and identified features of SS provision and receipt. Participants answered specific questions about the SS they received, including the type of provider of the SS (e.g. family member) who was subsequently referred to as the participant’s “SS partner.” Participants reported an average of 15.98 (*SD* = 5.10) episodes over the two days. SS receipt was reported in 692 total episodes by 163 different participants (*M* = 4.25, *SD* = 2.70). For the purposes of this study, we were especially interested in episodes involving SS receipt. However, all responses were included in analyses comparing episodes in which SS was and was not received.

### Measures

#### Psychological outcomes

At the beginning of each episode, participants indicated the extent to which they had experienced several single-item emotions or psychological outcomes during the episode, using a scale from 1 (*not at all*) to 7 (*very much*). Only stress and affection were considered for the purpose of this study. We considered both stress and affection in the previous same-day episode and current stress and affection. Specifically, we evaluated the extent to which participant country and SS receipt predicted next-episode affection and stress using next-episode stress and affection (t+1) as our dependent variables. The intraclass correlation coefficient (ICC) for stress (*M* = 1.90, *SD* = 0.786) was .29, and ICC for affection (*M* = 2.68, *SD* = 1.40) was .36.

#### SS receipt

This measure assessed whether SS was provided to the participant in each episode. Participants were asked to respond with Yes (1) or No (0) to the question: *During this episode*, *did you receive any social support from someone else*? SS was received in 24.4% of the episodes.

#### Esteem-building SS

This 4-item scale, adopted from Chen et al. [5], asked participants to rate how much they believed the SS they received was related to boosting self-esteem. Participants responded using a scale from 1 (*not at all*) to 7 (*very much*) to indicate how much they believed their SS partner boosted their self-esteem for each SS episode (*M* = 5.04, *SD* = 1.70). Example items included were: *My partner boosted my self-esteem* and *My partner helped me feel better about myself*. ICC = .42. Multilevel interitem consistency (α = .91) was estimated using the approach described by Nezlek [33], and for R by Bonito et al. [34].

#### Closeness-fostering SS

This 4-item scale, also adopted from Chen et al. [5], asked participants to rate how much they believed the SS they received was related to fostering closeness. Participants responded using a scale from 1 (*not at all*) to 7 (*very much*) to indicate how much they believed their SS partner fostered closeness during each SS episode (*M* = 5.06, *SD* = 1.74). Example items included: *The social support helped my partner and me feel close together* and *My partner helped me to know she/he cared about me*. ICC = .32, α = .94.

## Results

### Data analysis overview

Multilevel modeling was completed using R 3.6. Specifically, the lme4 [35], jtools [36], lmerTest [37], reghelper [38] and interactions [39] packages were used for the analyses presented in this section. Multilevel modeling was necessary because episodes (at Level 1) are nested within person (Level 2). Most variables of conceptual interest, including stress, affection, SS receipt, and closeness and esteem SS, were observed at Level 1 (L1). Individual differences between people, including differences in country (Singapore vs. U.S.) were measured at Level 2 (L2). In all cases, random between-person variability was first estimated, but was only retained in the model if it explained meaningful variability in the outcome (at *p* < .10). All continuous predictors at L1 were group mean centered prior to analysis. The dichotomous predictor of SS receipt was entered as a dummy coded variable at L1 and cultural context was considered at L2 (U.S. coded as 0, Singapore coded as 1). Identical sets of analyses were used to predict next-episode stress and next-episode affection from the indicators of SS receipt. Analyses used .05 as the threshold of significance.

Two major sets of analyses were undertaken. The first set made use of all available participants and episodes in the study. The goal of analyses was to determine whether episodes in which participants reported receiving SS were associated with more or less stress or affection than is typical for that participant, and whether these associations varied by country. The second set of analyses utilized only those episodes in which participants reported receiving SS. Specifically, we tested whether the esteem-building or closeness-fostering SS predicted next-episode stress and affection, and again tested whether those associations varied by cultural context.

Each model was built sequentially, beginning with a simple model that used only the L1 SS predictor (random or fixed, as appropriate) to predict the L1 outcome, as shown below for the SS receipt and stress analysis.

### Model 1

Level 1: Stress_ij_ = *π*_*0*j_ + *π*_*1j*_(SS Receipt_t-1_) + *e*_*ij*_

Level 2: *π*_*0j*_ = *β*_*00*_ + *r*_*0j*_

Next, the L2 moderator of cultural context was added to the model. We tested for cultural differences in the outcomes, as well as whether the effects of SS receipt were moderated by country. Please see the example formulas below.

### Model 2

Level 1: Stress_ij_ = *π*_*0*j_ + *π*_*1j*_(SS Receipt_t-1_) + *e*_*ij*_

Level 2: *π*_*0j*_ = *β*_*00*_ + *β*_*01*_(Country) + *r*_*0j*_

*π*_*1j*_ = *β*_*10*_ + *β*_*11*_(Country) + *r*_*1j*_

Finally, in Model 3, each test was conducted with the addition of the prior same-day episode’s stress (or affection) rating, so the focus of this final analysis is on the extent to which prior SS and cultural context predicted next-episode stress, above and beyond prior stress (or affection).

### Model 3

Level 1: Stress_ij_ = *π*_*0*j_ + *π*_*1j*_(SS Receipt_t-1_)+*π*_*2j*_(Stress_t-1_) + *e*_*ij*_

Level 2: *π*_*0j*_ = *β*_*00*_ + *β*_*01*_(Country) + *r*_*0j*_

*π*_*1j*_ = *β*_*10*_ + *β*_*11*_(Country) + *r*_*1j*_

*π*_*2j*_ = *β*_*10*_ + *r*_*2j*_

The results of all tests of Model 3 are shown in Table 1 and described below. However, the results of Model 1 and 2 were entirely consistent with those described here. All cultural differences identified in these analyses were also present when the prior same-day measure of stress (or affection) was excluded from the model. For parsimony, we omitted these results from the manuscript. Please also note that to reduce length and complexity, we report all estimated Model 3 parameters only in the table and focus on the simple slopes in the text.

**Table 1:** Parameter estimates from multilevel regression models predicting next-episode stress and affection from previous emotion as a function of SS receipt and country.

	Next-episode Stress		Next-episode Affection	
Predictor	*b* value (*SE*)	*p*	*b* value (*SE*)	*p*
Intercept	2.50*** (.09)	< .001	2.84*** (.12)	< .001
Receipt	-0.16 (.11)	.11	0.11 (.12)	.39
Country	0.36** (.14)	.01	0.71*** (.20)	< .001
Country x Receipt	0.39** (.14)	.01	-0.37* (.16)	.02
Prior Emotion	0.07** (.03)	.01	0.17*** (.03)	< .001
Intercept	2.47*** (.14)	< .001	3.12*** (.18)	< .001
Closeness	-0.04 (.07)	.51	0.29*** (.09)	< .001
Country	0.68*** (.19)	< .001	0.22 (.25)	.38
Country x Closeness	0.09 (.09)	.34	-0.35** (.11)	.01
Prior Emotion	0.11** (.05)	.01	0.12* (.05)	.02
Intercept	2.46*** (.14)	< .001	3.12*** (.18)	< .001
Esteem	-0.10 (.08)	.22	0.33** (.10)	.01
Country	0.70*** (.19)	< .001	0.23 (.25)	.37
Country x Esteem	0.24* (.10)	.02	-0.29* (.13)	.02
Prior Emotion	0.12** (.05)	.01	0.13** (.05)	.01

*Note*:

*p < .05

**p < .01

***p < .001

### Cultural differences

Initial analyses tested cultural differences in the frequency of reported SS receipt, and also in reports of stress, affection, and esteem and closeness SS. Specifically, those respondents from Singapore (*M* = 4.90, *SD* = 3.12) reported receiving SS in more episodes than did those in the U.S. sample (*M* = 3.05, *SD* = 2.29), *t*(176) = 4.55, *p* < .001. Further, two multilevel models predicting L1 stress and affection from country suggest that those in Singapore also reported feeling greater affection (*b* = 0.56, *t*(176) = 2.99, *p* = .01 and more stress (*b* = 0.44, *t*(176) = 3.22, *p* = .01) than did those in the U.S. However, participants in Singapore and the U.S. did not differ in their ratings of SS closeness (*b* = -0.03, *t*(156.46) = -0.13, *p* = .90) or esteem-building (*b* = -0.35, *t*(156.45) = -1.66, *p* = .09). These findings did not support our hypothesis that participants in Singapore would report more closeness and U.S. participants would report more esteem-building SS.

### Social support receipt

**Fig 1:**
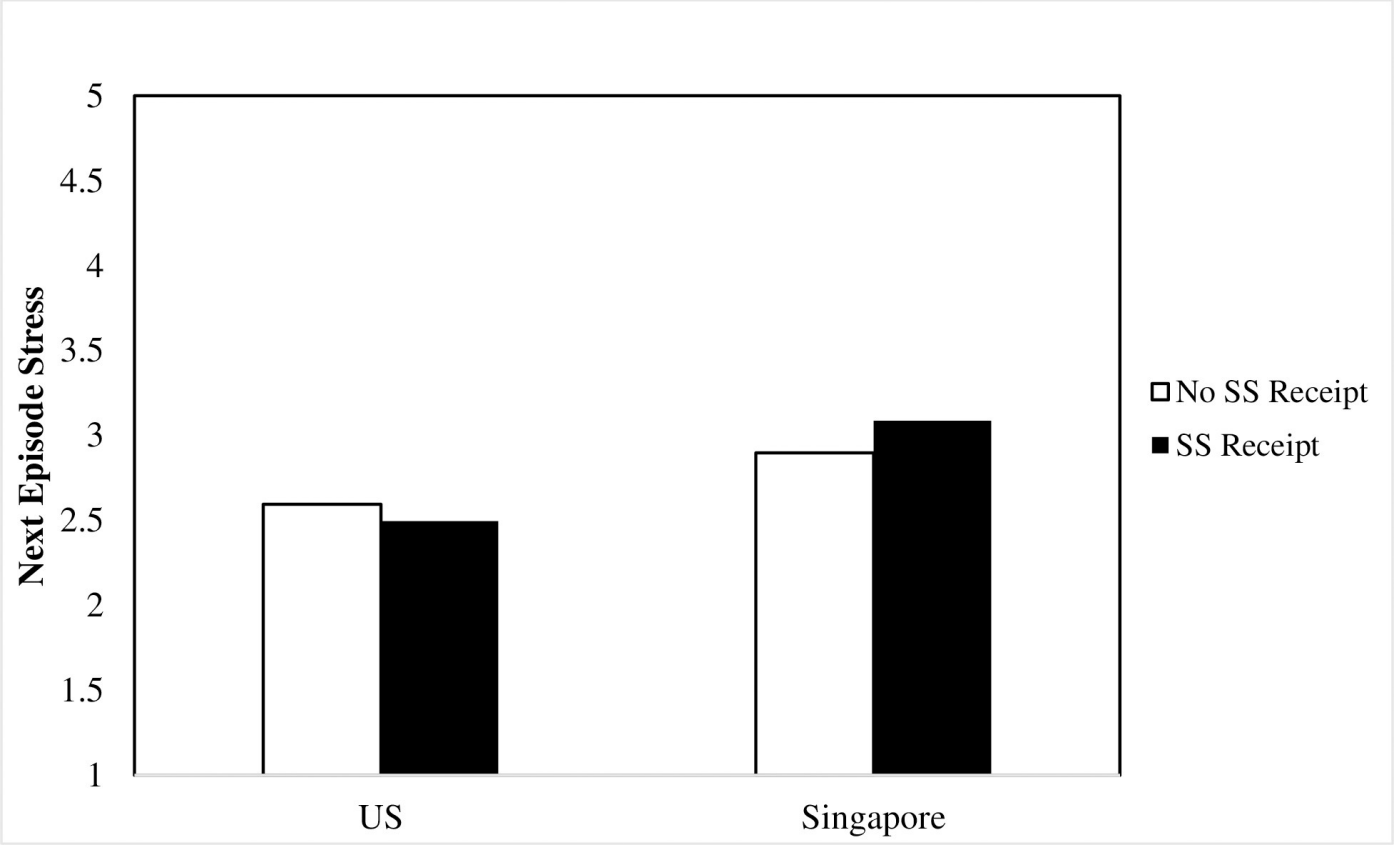
Interaction of culture and social support receipt on next episode stress.

As shown in Table 1, analyses did not suggest that episodes following SS receipt differed in reported stress but did suggest that the relationship between SS receipt and next-episode stress varied by country. Specifically, as summarized in Table 1, after accounting for the effects of prior stress on current stress, participant country moderated the effects of prior SS receipt on current stress. The results shown in Fig 1 suggest that although respondents from Singapore reported greater stress overall, that difference was especially pronounced following episodes in which they received SS (*b* = 0.24, *SE* = .10, *p* = .01), supporting our hypothesis that participants from Singapore would report more stress following SS receipt compared to participants in the U.S. Participants in the U.S. did not show elevated stress following SS receipt. In fact, the direction of the non-significant simple slope was negative (*b* = -0.15, *SE* = .10, *p* = .12).

A complementary pattern emerged when predicting next-episode affection from SS receipt. Analysis of simple slopes suggested that between-country differences in affection disappeared following SS receipt. Specifically, those in Singapore reported significantly lower affection following SS receipt (*b* = -0.37, *SE* = .12, *p* < .01), making those reports similar to affection reported in the U.S. In contrast, participants in the U.S. reported similar affection following episodes in which they did and did not receive SS (*b* = 0.02, *SE* = .12, *p* = .87).

### Closeness-fostering SS

As described above, an additional set of analyses were used to test the effects of closeness and esteem SS receipt. Specifically, reports of next-episode stress were predicted from prior episode SS recipient ratings of the belief that SS providers promoted closeness. As shown in Table 1, closeness-fostering SS did not predict next-episode stress, and that association was not moderated by country. These findings did not support our hypothesis that culture would moderate participants’ experience of stress following closeness-fostering SS.

In contrast, cultural differences were observed in the relationship between closeness-fostering SS and next-episode affection. Specifically, as summarized in Table 1, Singaporean participant reports of closeness-fostering SS did not predict next-episode affection (*b* = -0.06, *SE* = .08, *p* = .44). However, participants in the U.S. showed a strong association between closeness-fostering SS and next-episode affection (*b* = 0.29, *SE* = .09, *p* < .01). At times when U.S. respondents reported relatively more closeness-fostering SS, they also reported more feelings of affection in the next episode. These findings did not support our hypothesis that participants in Singapore would report greater feelings of affection after receiving closeness-fostering SS compared to participants in U.S., but did indicate cultural differences in the emotional correlates of closeness-fostering SS.

### Esteem-building SS

A final set of analyses examined the relationship of esteem building on next-episode stress and affection. Specifically, Table 1 indicates that reports of stress were not directly predicted from prior episode SS recipient ratings of esteem-building SS, but that association was moderated by country. Specifically, for respondents in Singapore, prior episode esteem-building SS predicted stress (*b* = 0.14, *SE* = .07, *p* = .05). In contrast, stress did not differ following esteem-building SS for participants in the U.S., (*b* = -0.10, *SE* = .08, *p* = .22). These findings supported our hypothesis that participants in Singapore would report relatively more stress following esteem-building in contrast to participants in U.S.

**Fig 2:**
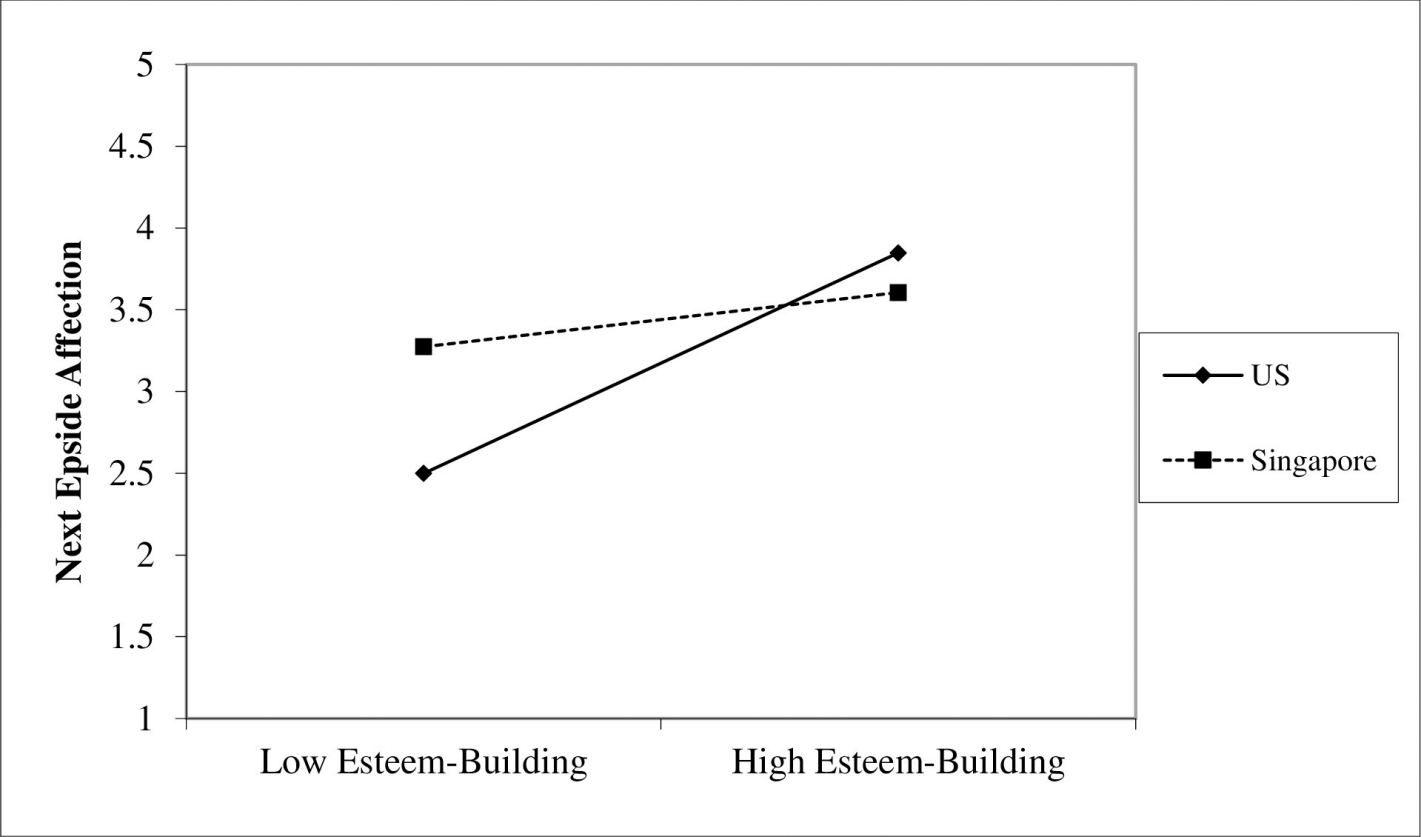
Interaction of culture and esteem building on next episode affection.

A complementary pattern emerged when examining cultural differences in esteem-building SS on next episode affection. Specifically, as seen in Fig 2, those in the U.S. showed a strong positive association between prior episode esteem-building SS and current affection, (*b =* 0.33, *SE* = .10, *p* < .001). In contrast, although those in Singapore reported greater affection overall, there was no effect of prior episode esteem-building SS on affection (*b* = 0.04, *SE* = .09, *p* = .68). These findings supported our hypothesis that participants in the U.S. would report more affection following esteem-building SS than to participants in Singapore.

## Discussion

The goal of the present study was to examine whether the psychological consequences of SS receipt for stress and affection would differ by cultural context, and whether receiving culturally appropriate SS was linked with later emotions. Specifically, we explored whether receiving esteem-building SS or closeness-fostering SS would predict next-episode stress and next-episode affection for SS recipients in Singapore and the U.S.

Results suggest that participants in Singapore reported more affection and stress than participants in the U.S., regardless of SS receipt. Participants in Singapore also reported receiving more SS than did participants in the U.S. In episodes following SS receipt, those in the U.S. reported more affection, whereas those in Singapore reported more stress.

Contrary to what we expected, there were no cultural differences in participant ratings of closeness-fostering SS or self-esteem-building SS. Previous cross-cultural research suggests that *providing* SS intended to foster closeness would be more culturally normative in an interdependent cultural context such as Singapore, while providing SS intended to build self-esteem would be more culturally normative in an independent cultural context such as the U.S. [5, 9]. However, when examining SS interactions, research shows that the experiences of the SS recipient may differ from the experiences of the SS provider [7]. Because the present study measured SS recipients’ perceptions of esteem or closeness SS they received, it is possible that SS providers were, in fact, providing one form of support, but the recipients believed instead that they were receiving another form of support (e.g., esteem-building SS). Even within a shared cultural context, perceptions of SS motivation can differ between the provider and the recipient [7]. Such ambiguous or forms of SS may result in differing perceptions of SS motivation between the provider and the recipient [7]. This study did not include complimentary reports from SS providers, so we are unable to assess this possibility. It is important to recognize that depending upon the perspective and situational lens through which SS is examined, SS motivation may be regarded differently for the SS provider than for the SS recipient.

Additionally, results indicate that country moderated the effects of prior SS receipt on next-episode stress. Participants in Singapore reported more stress following episodes in which they received SS, but this pattern was not present for participants in the U.S. These findings are consistent with previous research suggesting that people in more interdependent cultural contexts are more likely to experience negative emotions following SS receipt [2]. These negative emotions can be the result of feeling a sense of shame or burden for requiring SS or inconveniencing the SS provider [10, 40]. Receiving SS could be a relatively stressful experience for people in more interdependent cultural contexts like Singapore.

Similar patterns emerged for examinations of the effects of country and prior SS receipt on next-episode affection. Specifically, participants in Singapore reported lower affection following episodes where they received SS. This reduction in affection after receiving SS resulted in relatively similar affection levels among both U.S. and Singaporean participants. One potential reason why participants in Singapore reported lower levels of affection after receiving SS could be that they could feel burdensome to their SS partner, resulting in feelings of shame or other negative emotions [40] that may hinder their feelings of affection. These effects could be compounded over time through processes such as rumination. Furthermore, participants in the U.S. reported similar affection regardless of whether they received SS. Those in more independent cultures tend to value the self [11], and therefore individuals in the U.S. may have been preoccupied with their current stressor and not focused on feelings of affection toward their SS provider.

Surprising patterns emerged in examining participants’ perceptions of SS receipt. *Closeness-fostering* SS did not predict next-episode stress and this association was not moderated by country. However, the relationship between closeness-fostering SS and next-episode affection did vary by country. Participants in the U.S. reported greater next-episode feelings of affection after reporting SS with relatively more closeness, but no association between closeness-fostering SS and next-episode affection was found for participants in Singapore. This cultural difference is in the opposite direction than expected. It is possible that, in contrast to the thought process informing our original predictions, in more interdependent contexts like Singapore, interpersonal closeness is seen as an obligatory component of group connection [11]. If closeness with others is considered a commonplace phenomenon, SS interactions may not elicit particularly pronounced feelings of affection in the SS recipient. Instead, the SS recipient may perceive the interaction to be the SS provider fulfilling their duty in the social relationship. We were surprised to see that closeness-fostering SS did predict later feelings of affection for participants in the U.S. This finding is inconsistent with a similar study of couples in the U.S. Gleason et al. found that SS receipt predicted negative emotions and feelings of *closeness* (they did not examine affection) [17]. The participants in the previous study were cohabitating adult couples, whereas our participants were college students with a wide range of SS providers including family members, friends, and classmates. More research is needed to further explore the ways in which relationship to the SS provider may influence emotional correlates of SS interactions.

Another potential explanation for the fact that closeness-fostering SS did not predict affection in Singapore may have the way that Singaporean participants conceptualized the item “affection.” It may be that in some interdependent cultures the feeling of affection can be seen as a component of love, and that in those cultures, relatively less value is placed on love or the notion of love as it is interpreted in Westernized cultures [41]. Those in Singapore may not have felt as positively after the fostering of closeness because affection may not be considered as important as other psychological outcomes in Singapore. In contrast, in more independent cultural contexts like the U.S., fostering closeness and connection may not be emphasized as highly overall. Participants in the U.S. may therefore have felt especially affectionate toward their SS partners for going above and beyond what was expected of them in the context of a social interaction.

Additionally, we examined cultural differences in the relationship between *esteem-building* SS and next-episode stress. For participants in Singapore, esteem-building SS was associated with relatively more next-episode stress. This is consistent with previous research suggesting that certain types of SS, such as emotion focused SS, may be culturally inappropriate in more interdependent cultural contexts [5]. Participants in interdependent cultural contexts who receive SS intended to boost self-esteem may face loss of respect or potential humiliation, leading to more negative psychological outcomes such as stress [42]. Similarly, cultural differences in the spillover of negative affect following SS may be facilitated through repetitive thought or rumination. Importantly, prior research suggests that Asian and Asian Americans report more rumination than European Americans [43], and that in comparison to more independent cultural contexts, those from more interdependent cultural contexts tend to be more self-critical [44]. Taken together, these patterns may suggest that participants in Singapore could have felt self-critical about requiring esteem-boosting SS, and rumination over the experience could have compounded stress over time, resulting in a spillover of negative emotions from one episode to the next. However, for participants in the U.S., esteem-building SS did not predict next-episode stress. This was unexpected considering previous research suggests that higher self-esteem is associated with greater well-being for individuals in more independent cultures [10]. One explanation for our lack of significant findings could be that participants in the U.S. may be somewhat desensitized to esteem-building support. Because SS designed to boost self-esteem is considered culturally normative in an independent cultural context such as the U.S. [5], it is possible that esteem-boosting from SS partners is so commonplace that the spillover from the prior esteem-building SS has little to no effect on stress. Conversely, lower self-esteem could be masking the effects of esteem-building SS. If participants’ self-esteem was leading to feelings of inefficacy [17], it is possible that these feelings could be counteracting the benefits of esteem-building.

Finally, a complementary pattern emerged for *esteem-building* SS and next-episode affection. For participants in the U.S., perceptions of esteem-building SS receipt predicted relatively more next-episode affection. In contrast, there was no effect of esteem-building SS receipt on next-episode affection for participants in Singapore. These patterns are expected in the U.S., as esteem-building is a culturally normative form of SS in that cultural context [5]. Previous research supports this pattern, such that people in independent cultural contexts with greater self-esteem reported higher affection expressions toward their romantic partner than those with low self-esteem [45]. Perhaps participants in the U.S. may feel more affectionate after they believed their SS provider tried to build their self-esteem because they actually did feel better about themselves, resulting in more positive affection overall.

### Limitations and future directions

The results of this study should be considered in light of some limitations. First, we assessed SS recipients’ perceptions of the SS without knowing the provider’s actual motivations for providing that SS. Although the recipient’s perspective is valuable, it may not show the full picture of the SS interaction. Likewise, we do not have any information about the content of any actual SS transactions between provider and recipient or know whether those interactions actually occurred. It is possible that SS recipient perceptions may be affected by many factors, including their overall impression of their SS partner, their satisfaction with the outcome of the interaction, or the responsiveness of the SS provider. If SS recipients do not perceive their SS partner to be responsive to their needs, even if the SS provider believes they are providing sufficient SS, SS may not benefit the recipient [9]. The SS recipient’s perspective may allow us to better understand the benefits of SS for emotion and health outcomes.

Additionally, all of the measures used for this study were self-report. Although DRM is designed to reduce memory bias and increase emotional recollection [46], it is not impervious to subjectivity and biases in reports based on faulty memory or other inaccuracies in reporting. Further, the measures of stress and affection were rated through only one item. Although single item responses decrease participant burden, especially over the repeated DRM assessments, they can be subject to error.

Future research might further consider how the conceptualization of psychological outcomes such as stress and affection differ across cultures. As this study shows, these emotions operate differently in the more interdependent cultural context of Singapore than in the more independent context of the U.S. Therefore, it is important to define emotions relative to the culture in which they are being studied. For example, because affection may be conceptualized and understood differently in a more interdependent culture [41], it should be examined in a cultural context. It is unlikely that all emotions are valued identically cross-culturally [47]. Not only is it important to consider the role of culture in the study of stress and affection, but it is also important to explore how culture may influence the expression and experience of other emotions such as shame [48] and pride [49], especially in socially supportive interactions. Cultural differences should therefore be considered in future research on SS and emotions.

Future research should consider cultural differences in the short- and long-term consequences of emotional responses to SS provision. SS receipt appears to have lingering emotional consequences. Future research might also test reversed or longer micro-longitudinal sequences of SS exchanges. For example, stress in episode 1 may predict SS in episode 2, which in turn could predict increased affection and decreased stress in episode 3. These questions might be especially well-suited to an ecological momentary assessment study that examines emotion in cross-cultural contexts. These techniques capture the complex interpersonal dynamics of SS provision and receipt, as well as how those dynamics unfold over short time periods [32]. Such patterns may not be apparent in laboratory settings. Experience sampling also allows participants to report memories that are episodic in nature, so the emotional consequences are less affected by retrospective recall biases, allowing true cultural differences between participant groups to be captured [50].

Overall, the current study highlights the importance of culture for understanding SS processes, and of examining the lingering consequences of SS on recipient stress and affection. Because culture influences many aspects of socially supportive interactions, it stands to reason that participants’ psychological outcomes following SS receipt would also differ by cultural context. This study also supports the current literature on cultural differences in esteem-building and closeness-fostering in SS interactions and extends it by examining recipient perceptions and emotions. In sum, the current study provides evidence that cultural differences emerge in SS processes and psychological outcomes of SS receipt. These patterns have implications for how socially supportive relationships can help to protect against the negative consequences of stress and promote health.

## Abbreviations

SS: social support
